# Species and abundance of ectoparasitic flies (Diptera) in pied flycatcher nests in Fennoscandia

**DOI:** 10.1186/s13071-015-1267-6

**Published:** 2015-12-21

**Authors:** Tapio Eeva, Tommi Andersson, Åsa M. M. Berglund, Jon E. Brommer, Raimo Hyvönen, Tero Klemola, Toni Laaksonen, Olli Loukola, Chiara Morosinotto, Kalle Rainio, Päivi M. Sirkiä, Eero J. Vesterinen

**Affiliations:** Department of Biology, University of Turku, FI-20014 Turku, Finland; Kevo Subarctic Research Institute, University of Turku, FI-20014 Turku, Finland; Department of Ecology and Environmental Science, Umeå University, Umeå, Sweden; Varnankatu 2 D, FI-20320 Turku, Finland; Department of Biology, University of Oulu, FI-90014 Oulu, Finland; Finnish Natural History Museum, Zoology Unit, University of Helsinki, FI-00014 Helsinki, Finland; Department of Agricultural Sciences, Spatial Foodweb Ecology Group, University of Helsinki, FI-00014 Helsinki, Finland

**Keywords:** Blood parasites, Bird blowflies, Ectoparasite prevalence, Louse flies, Pied flycatcher

## Abstract

**Background:**

Birds host several ectoparasitic fly species with negative effects on nestling health and reproductive output, and with the capability of transmitting avian blood parasites. Information on the abundance and distribution of the ectoparasitic fly genera *Ornithomya* (Hippoboscidae) and *Protocalliphora* (Calliphoridae) in northern Europe is still generally poor, and we thus explored their geographic range and occurrence of these flies in the nests of a common avian model species, the pied flycatcher *Ficedula hypoleuca*.

**Methods:**

Nests of *F. hypoleuca* were collected from 21 locations across Fennoscandia in summer 2013, across a latitudinal gradient (between 56 °N – 70 °N) and examined for the presence of fly puparia. Adult specimens of *Ornithomya* spp. were also collected for species identification. Fly species were identified morphologically and identifications confirmed with DNA barcoding.

**Results:**

We found three species: two louse-flies − *Ornithomya chloropus* and *O. avicularia* − and one blow-fly, *Protocalliphora azurea*. The prevalence of *O. avicularia* was higher in southern latitudes and this species was not encountered beyond 62 °N whereas *O. chloropus* and *P. azurea* occurred across the whole range of latitudes. The prevalence of *O. chloropus* further increased with increasing distance from the coast – a pattern not documented before. The three fly species showed no interspecific associations in their prevalence.

**Conclusions:**

Our study revealed relatively high prevalence for all the species (*O. chloropus* 59 %, *O. avicularia* 20 %, *P. azurea* 32 %), and an interesting spatial pattern in the prevalence of the two louse fly species. Our sample did not indicate any major range shifts towards the north for the southern species as compared to the information from the past. Morphological identification of *O. chloropus* did not match with the corresponding sequences published in the GenBank and taxonomy of this group calls for further studies.

**Electronic supplementary material:**

The online version of this article (doi:10.1186/s13071-015-1267-6) contains supplementary material, which is available to authorized users.

## Background

Ectoparasitic louse flies *Ornithomya* spp. (Diptera, Hippoboscidae) and bird blowflies *Protocalliphora* spp. (Diptera, Calliphoridae) commonly infest nestling passerines, the former ones sucking blood from nestlings as adults and the latter as in their larval stage [1–3]. Both parasites have been found to inflict negative effects on nestlings, though their effect on nestling mortality is usually weak and causality between parasite numbers and mortality or condition often remains unclear [4–9]. However, louse flies are vectors of some avian blood protozoans (e.g. *Haemoproteus* spp. and *Trypanosoma* spp.), and could have delayed effects on condition or mortality [10–12]. Sub-lethal effects of parasitic bird blowflies include anemia and retardation of growth [5–8, 13]. In Fennoscandia, four species of *Ornithomya* [14] and five species of *Protocalliphora* [15] occur but the information on distribution and abundance of these species are incomplete.

Ectoparasites – not only flies but also ticks, mites, and fleas – may also host and transmit zoonoses between animal species, and from animals to humans, including e.g. migratory birds [16], birds generally [17, 18], raccoon dogs [19], and bats [20, 21]. Especially urban areas have been studied in detail, due to the higher risk for human population [22]. Thus, it is important to identify ectoparasites, reveal their geographical distribution, and unveil ecological interactions with their hosts.

We studied the occurrences of *Ornithomya* and *Protocalliphora* species in nests of a common avian model species, the pied flycatcher *Ficedula hypoleuca* Pallas 1764 across a latitudinal gradient between 56 °N – 70 °N in Fennoscandia. The relationships between *Ornithomya* and *Protocalliphora* prevalence and breeding success of *F. hypoleuca*, as well as the dependence of parasite prevalence on some environmental variables (e.g. temperature, biotope and pollution) were explored in an earlier paper [9] and are not dealt with here. We identified the fly species on the basis of morphological characters of their puparia (both genera) and adult specimens (for *Ornithomya*). Some specimens were further DNA barcoded to confirm the morphological identification. Since both fly genera are favored by increasing summer temperatures [9] we were also interested to find out if some of the southern fly species have spread towards the north during the recent period of warming climate in Fennoscandia [23]. We further tested for the association of prevalence between different fly species in parasitizing *F. hypoleuca* broods, i.e. whether their occurrence in a nest is negatively (e.g. by competition; [24]) or positively (e.g. by host quality) associated.

## Methods

**Fig. 1:**
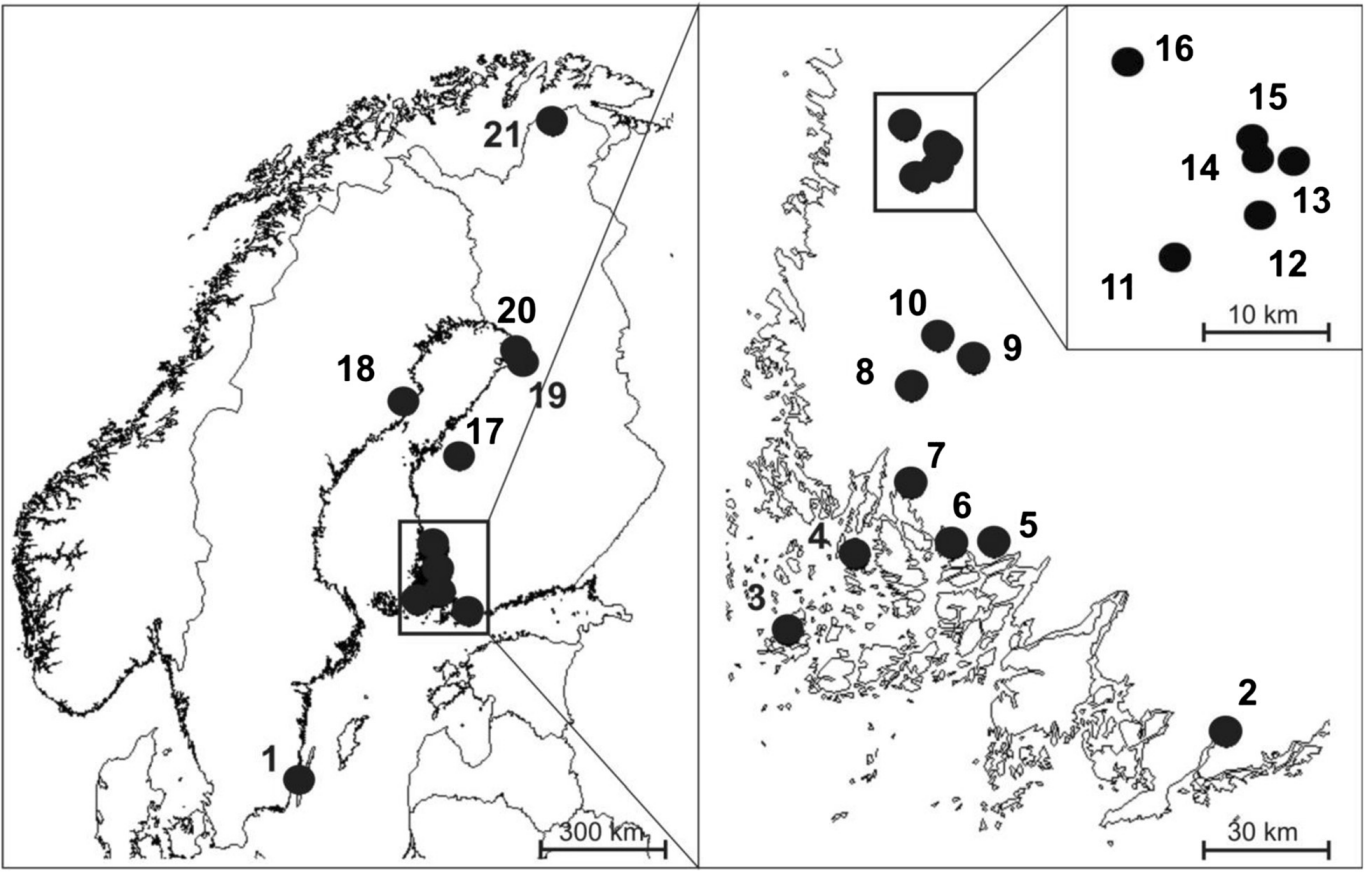
Maps of Fennoscandia showing 21 locations where the 236 *Ficedula hypoleuca* nests were collected for this study. More detailed maps are shown for SW Finland, and sites in Satakunta

A total of 236 nests of *F. hypoleuca* were collected from 21 locations in summer 2013 and parasites recovered and counted (Table 1 and Fig. 1). Only successful nests, i.e. where at least one nestling finally fledged, were sampled. All nest boxes were emptied before the breeding season. All the nest material (including dust at the bottom) was carefully removed from the wooden nest boxes usually within one week of fledging, stored in plastic bags and frozen until the samples were inspected in a laboratory for the puparia of *Ornithomya* and *Protocalliphora*. Adult *Ornithomya* feed especially on nestlings and deposit one fully-grown larva at time [1]. The larvae immediately pupate, overwinter, and hatch the following season [1]. Larvae of *Protocalliphora* feed periodically on the blood of nestling birds and pupate in the nest material [15, 25]. The adults emerge some weeks later, overwinter, and lay their eggs following season after nestlings hatch [3, 15].

**Table 1:** Sample locations, their distance from the coast and collection dates for *Ornithomya chloropus*, *O. avicularia* and *Protocalliphora azurea* in *F. hypoleuca* nests in summer 2013 (*N* = no. of nests in sample, *P* = prevalence [% nests infested], (*n*) = no. of infested nests, I = intensity [mean number of puparia per infested nest])

							*O. chloropus*		*O. avicularia*		*P. azurea*	
N.o.	Location	Lat (°N)	Lon (°E)	Dist. to coast (km)	Dates	*N*	P % (*n*)	I	P % (*n*)	I	P % (*n*)	I
1	Borgholm	56.70	16.55	3.32	26.-28.6.	10	0	.	30 (3)	1.00	0	.
2	Raasepori	60.02	23.52	1.57	28.6.-2.7.	10	20 (2)	2.00	10 (1)	2.00	60 (6)	6.50
3	Houtskär	60.24	21.36	0.05	10.8.	3	0	.	33 (1)	3.00	100 (3)	5.67
4	Kaidanpää	60.41	21.70	0.04	2.7.	10	0	.	20 (2)	2.00	40 (4)	5.50
5	Littoinen	60.44	22.37	4.61	13.8.	3	0	.	0	.	0	.
6	Ruissalo	60.44	22.17	0.36	5.7.	10	0	.	70 (7)	2.29	40 (4)	6.75
7	Lemu	60.57	21.97	3.29	25.6.-14.7.	10	10 (1)	1.00	40 (4)	1.25	60 (6)	3.67
8	Lemmi	60.79	21.97	18.6	3.7.	12	75 (9)	3.44	8 (1)	1.00	58 (7)	3.00
9	Vaskijärvi	60.84	22.28	33.0	26.6.-5.7.	13	38 (5)	2.60	8 (1)	1.00	38 (5)	5.80
10	Karjala	60.90	22.10	32.5	29.6.	10	80 (8)	2.63	0	.	30 (3)	4.67
11	Panelia	61.25	22.00	21.0	8.7.	10	90 (9)	3.67	20 (2)	1.50	60 (6)	4.00
12	Palokangas	61.27	22.11	27.1	8.7.	20	80 (16)	5.00	10 (2)	1.00	20 (4)	1.75
13	Paloasema	61.31	22.14	29.7	1.7.	11	54 (6)	2.33	18 (2)	2.00	0	.
14	Ojala	61.31	22.11	28.2	1.7.	11	100 (11)	2.91	36 (4)	1.00	0	.
15	Koivula	61.31	22.11	28.0	1.7.	22	95 (21)	2.76	18 (4)	1.50	0	.
16	Kallioaro	61.36	21.94	19.6	8.7.	14	43 (6)	2.50	0	.	29 (4)	9.50
17	Kauhava	63.13	23.10	40.8	1.7.-9.7.	18	83 (15)	6.07	0	.	61 (11)	9.91
18	Umeå	64.20	20.85	7.29	1.7.-10.7.	10	60 (6)	3.67	0	.	20 (2)	4.00
19	Sanginjoki	65.02	25.77	14.4	5.7.	14	86 (12)	4.25	0	.	43 (6)	5.67
20	Kalimenkylä	65.12	25.51	6.40	2.7.	9	89 (8)	3.38	0	.	11 (1)	8.00
21	Kevo	69.76	27.01	69.0	8.7.-23.7.	6	67 (4)	2.50	0	.	17 (1)	7.00
Total						236	59 (139)	3.62	14 (34)	1.59	32 (76)	6.34

The puparia of *Ornithomya* were sorted out in two groups based on their morphological characters (size and surface structure) by using stereo microscope. Ten puparia of each type were further used for non-destructive DNA extraction and sequencing [26] and successful PCR products were purified and subsequently sequenced [27]. Whole specimens were incubated 24 h, then the puparia were removed and DNA was purified using either NucleoSpin® Tissue XS (small samples) or NucleoSpin® Tissue (all other samples) Kit (product numbers 740901 and 740952, respectively; Macherey-Nagel GmbH & Co. KG, Düren, Germany) from remaining solution. Adult specimens (*n* = 71) of *Ornithomya* were also collected from nestlings of *F. hypoleuca*, great tit *Parus major* L. and blue tit *Cyanistes caeruleus* L. and used for reference material to identify species morphologically and genetically. These came partly from the same nests (*n* = 19) of *F. hypoleuca* where the puparia were sampled. Adult specimens were identified using morphological characters by Antti Haarto (University of Turku). Some specimens (*n* = 18) were then used for DNA extraction and sequencing as described above. Only some of the specimens produced readable DNA sequences, with most of the failed specimens being puparia.

The species identity of all sequenced material was confirmed by downloading all available unique *Ornithomya* and *Protocalliphora COI* (*cytochrome oxidase subunit I*) DNA barcodes from GenBank and BOLD (The Barcode of Life Data Systems), aligning them with our own sequences, calculating distances between each sequence, and finally drawing both neighbor-joining (NJ) and maximum-likelihood (ML) consensus trees using software Geneious and plugins as follows. NJ tree was built with built-in Geneious tree builder with default settings except resampling was carried out with 100 bootstrapping replicates [28]. ML tree was built with PhyML plugin with default settings except for resampling with 100 bootstrap replicates [29].

We concentrate here in analyzing the spatial variation in prevalence because the different parameters showed relatively strong positive correlations (r_s_ between 0.50–0.98 in most cases). Furthermore, the number of parasites per nest is likely to be influenced by several environmental factors (e.g. temperature, biotope, brood size, host population density), as well as handling of nestlings [30], which were not controlled for in our study. Geographical trends in prevalence were analyzed for each species with generalized linear mixed model (GLMM; [31]) where occurrence (1 = found; 0 = not found) of a species was used as a binary response variable and latitude, longitude and logarithmic (base 10) distance from the coast were used as explanatory variables. Distance from the coast was added in the model as *ad hoc* variable after noticing in the field that in and close to the archipelago at SW Finland the relative proportions of two *Ornithomya* species were different than in the inland. Location was used in the models as a random effect to control for spatial independence of the observations. Degrees of freedom were calculated with Kenward-Roger method. The level of significance was set at *p* < 0.05. Non-significant terms were dropped out from the models one by one, starting from the least significant term. The residuals from all models were further tested for spatial autocorrelation with Moran’s I coefficients. These indicated no significant spatial autocorrelation in our data (range for I: −0.0099 to 0.0006; *p* > 0.45 in all). For *Ornithomya*, the reduced models were also run without the cases (*O. chloropus*, *n* = 16; *O. avicularia*, *n* = 3) where some adult flies were collected away from host nests (because this could affect the number of puparia). Since this did not markedly affect the results we report the models with full data.

The associations in occurrence between the two observed *Ornithomya* species and between *Ornithomya* and *Protocalliphora* were tested with *χ*^2^ test in a contingency table with frequencies of occurrence (1 = found; 0 = not found). For the analysis testing an association between the *Ornithomya* species we only included the nests from those study sites (*n* = 9; Table 1) where both species were found. This was because testing such associations outside the range of the species would not be meaningful.

## Results

On the basis of morphological identification all adult *Ornithomya* specimens (*n* = 71) collected from the nests belonged to two species, *O. chloropus* Bergroth 1901 and *O. avicularia* L. The adult specimens used for genetic identification also formed two clearly separate clusters confirming our identification accuracy. However, the species names assigned for the GenBank sequences for the specimens morphologically identified as *O. chloropus* clustered together with a sequence labeled as *O. anchineura* Speiser 1905 (match 98.3–98.9 %; a taxon described from North America; GenBank accession number EF531227). However our *O. avicularia* shared highest identity with the *O. avicularia* sequence in GenBank (match 99.1–100 %; EF531211 and KF453421; see Additional file 1 for taxonomic tree and Additional file 2 for distance table).

**Fig. 2:**
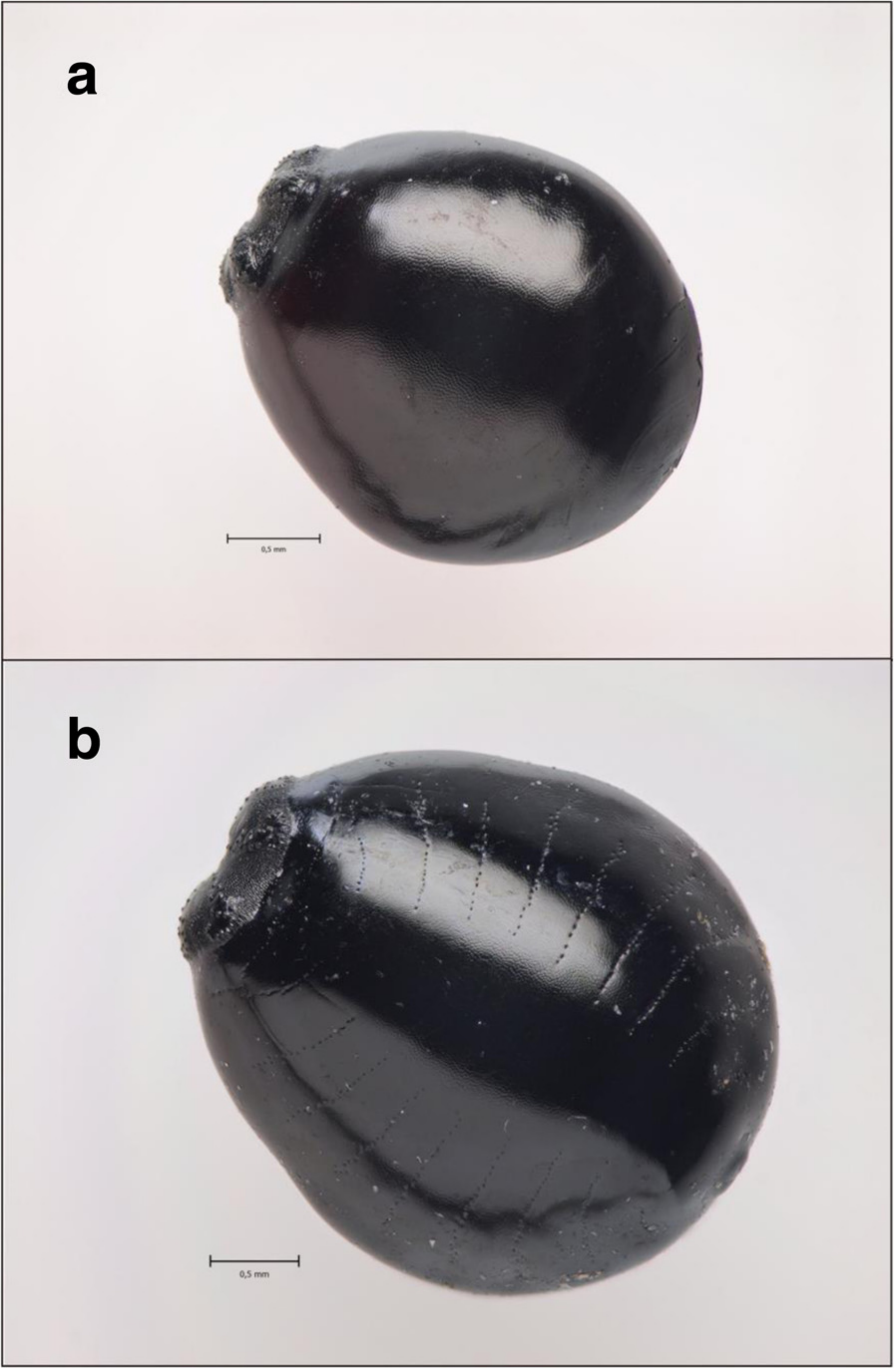
Puparia of *Ornithomya chloropus* (**a**) and *O. avicularia* (**b**). Note the larger size and prominent dotted lines of *O. avicularia*. Scale lines 0.5 mm. Photo: Veikko Rinne

The puparia of *Ornithomya* were also morphologically identified to form two groups, the larger ones *O. avicularia* (larger species), and the smaller ones as *O. chloropus* (smaller species) (Fig. 2). The molecular results confirmed our identification into two clearly separate species (Additional file 1). However, as in the case of adults, the sequences of puparia clustered with *O. anchineura* and *O. avicularia*. As we currently have no knowledge of the true taxonomical status of the *O. anchineura* specimen in the GenBank, we retain to our own morphologically identified species names in this study, that is, *O. chloropus*, and *O. avicularia. Protocalliphora* puparia were determined as *P. azurea* Fallén 1817 (GenBank accsession CQ409352).

**Fig. 3:**
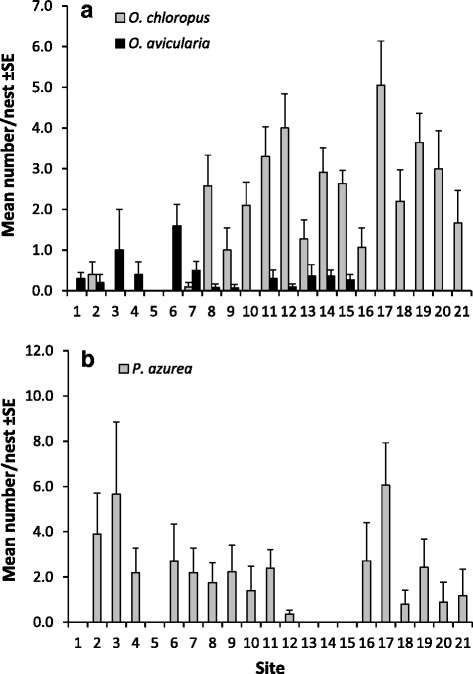
Means and standard errors for the numbers of (**a**) *Ornithomya* species and (**b**) *Protocalliphora azurea* puparia in the nests of *F. hypoleuca* at sampling sites (sorted from south to north; see Fig. 1). Sample sizes are shown in Table 1

**Fig. 4:**
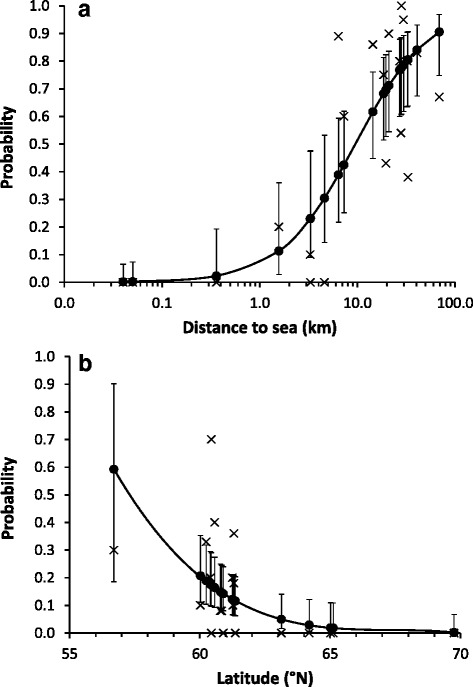
Prevalence (×) in *F. hypoleuca* nests of (**a**) *O. chloropus* relative to distance from the coast, and of (**b**) *O. avicularia* relative to latitude. Predicted values (●) and 95 % confidence limits are produced by reduced models shown in Table 2. Sample sizes are shown in Table 1

Mean prevalence and intensity for all locations are given in the Table 1 and the mean numbers of puparia per nest are shown in the Fig. 3. The prevalence of *O. chloropus* (mean 59 %) increased with increasing distance from the coast (Fig. 4a) but showed no significant latitudinal or longitudinal trend (Table 2). Instead, the prevalence of *O. avicularia* (below 61° the mean was 20 %) was higher in the south (Fig. 4b) but was not associated with the longitude or distance from the coast (Table 2). Because we had only one data point below 60° the predicted level for the southernmost location is very inaccurate (Fig. 4b). Latitudinal trend, however, was significant (GLMM, F_1,45.2_ = 5.1, *p* = 0.030) even when omitting the southernmost location from the analysis. The prevalence of *P. azurea* (mean 32 %) showed no significant geographical trend in our sample (Table 2).

**Table 2:** Generalized linear mixed models (GLMM^a^) for explaining geographical trends in prevalence of puparia of three parasitic fly species in the nests (*n* = 236) of *Ficedula hypoleuca*

	*O. chloropus*			*O. avicularia*			*P. azurea*		
	β ± SE	F _df_	*P*	β ± SE	F _df_	*P*	β ± SE	F _df_	*P*
Intercept	**−2.57 ± 0.85**			**29.7 ± 12.2**			**−0.422 ± 0.44**		
Latitude (°N)	−0.0153 ± 0.23	0.00 _1,12.0_	0.95	**−0.517 ± 0.20**	**6.61 **_**1,23.8**_	**0.017**	−0.167 ± 0.24	0.50 _1,15.0_	0.49
Longitude (°E)	0.237 ± 0.18	1.81 _1,20.3_	0.19	0.190 ± 0.35	0.30 _1,22.9_	0.59	0.200 ± 0.17	1.33 _1,18.9_	0.26
Log distance from the coast (km)	**2.63 ± 0.67**	**15.2 **_**1,26.6**_	**0.0006**	−0.432 ± 0.34	1.63 _1,15.2_	0.22	**−0.558 ± 0.35**	**2.48 **_**1,16.1**_	**0.13**

Reduced models are shown in bold

^a^GLMM with binary error distribution, logit link function, and location (*n* = 21) as a random factor

*Ornithomya chloropus* and *O. avicularia* did not show negative or positive association in their occurrence (*χ*^2^ = 0.006, df = 1, *p* = 0.95, *n* = 119 nests). Neither was there any association in the occurrence of *Ornithomya* spp. (two species pooled) and *P. azurea* (*χ*^2^ = 0.72, df = 1, *p* = 0.40, *n* = 236 nests).

## Discussion

From the nests of *F. hypoleuca,* we found two species of louse flies, *O. chloropus* and *O. avicularia*, and one bird blowfly species, *P. azurea*. The occurrence of a third common louse fly species in southern Finland, *O. fringillina* Curtis 1836, generally takes place later in the autumn (peaking in September and October) [14, 32] and was not found in *F. hypoleuca* nests, though we could not confirm the species for all the puparia collected with barcoding. Nor did we observe the most southern *Ornithomya* species in Fennoscandia, *O. biloba* Dufour 1827. Hill *et al*. [14] explored the Fennoscandian museum specimens of *Ornithomya* sp. (adults). *O. chloropus* showed wide distribution from Denmark to northern Norway (70 °N to 71 °N) whereas the northernmost specimens of *O. avicularia* were found between latitudes 60 °N − 61 °N (though occasionally reported farther north; [33]). Our study revealed similar pattern across latitudes, *O. chloropus* being found from 60.0 °N to 69.8 °N, while northernmost individuals of *O. avicularia* were found at 61.3 °N. This suggests that the southern species, *O. avicularia*, has not markedly expanded its range towards north since times before 1964 (time range for the museum samples was not given) despite that climate in Finland has been warming >2 °C since 1800′s (especially after 1960′s and especially at northern latitudes; [23]) and despite the abundance of *Ornithomya* spp. in *F. hypoleuca* nests is higher in warm summers [9]. However, due to relatively coarse latitudinal coverage of our study, as well as that of Hill *et al*. [14], small range shifts would likely be undetected with this comparison.

Both species of *Ornithomya* are hosted by many bird species [14, 34, 35] and since their host preferences are not well known the numbers found in the nests of *F. hypoleuca* may not be used as comparable estimates of their total abundance. For example, *O. avicularia* has been found to prefer relatively large host species (e.g. Turdidae and Corvidae) while *O. chloropus* accepts larger array of hosts with more variable size [36]. The general abundance of the former species could therefore be underestimated on the basis of *F. hypoleuca* nest material. In any case, on the basis of numbers and prevalence, *O. chloropus* poses higher parasitic stress on *F. hypoleuca* nestlings than *O. avicularia*, though even their combined intensity would have relatively weak acute effects on nestling survival [9]. In general, *Ornithomya* prevalence shows considerable annual variation, and is highest in warm summers [9]. In 2013, the weather during the main nestling period of *F. hypoleuca* was relatively warm (mean June temperature 16.5 °C in SW Finland) and the combined prevalence of the two *Ornithomya* species in SW Finland was higher than reported in the preceding years 2006–2012 [9]. Therefore, the prevalence levels found in the current study represent favorable conditions and likely differ from those found during colder summers.

We do not know why *O. chloropus* becomes less prevalent towards the coast, but the reasons could be climatic as the sea has a cooling effect on the coast during the spring [37] and the proximity of sea could delay the phenology of this parasitic fly. For *Lipoptena cervi* L., a louse-fly ectoparasite of cervids, low summer temperatures were found to prolong the developmental periods and delay the emergence of adult flies [38]. In our study area, also spatial heterogeneity of habitats is likely higher near the coast, forested areas farther from the coast being larger and more uniform. *O. chloropus*, however, is hosted by wide variety of bird species, including ones in open habitats [35], and is therefore not restricted to occur in forested areas. We found no association in prevalence between the two *Ornithomya* species and interspecific competition between them is an unlikely explanation for the observed spatial pattern. The observed pattern has not been reported earlier and it calls for further studies.

One successfully DNA barcoded *Protocalliphora* puparia was identified as *P. azurea*. On the basis of the similar morphological appearance of the rest of the puparia in our sample, we assumed all were the same species, though there still remains a possibility that some other species are included in our sample. However, in a sample of puparia grown to adulthood (*n* = 107 individuals), collected at or near the locations 10–16 (Fig. 1) in the beginning of the 1990s, no other species were found [39]. *Protocalliphora azurea* is widely distributed in the Palearctic region and is hosted by multiple bird species [15]. We found no significant geographical trends in the numbers of *Protocalliphora* puparia. Sites 13–15 are affected by air pollution from a copper smelter and scanty field layer vegetation may explain why no puparia were found at these locations since adult flies feed on flowers [25] and favor locations with luxuriant field layer vegetation [39]. A recent study from SW Finland found no effects of *Protocalliphora* on *F. hypoleuca* nestling mortality at corresponding levels of intensity as in the current study [9], but high intensities have been shown to increase nestling mortality in the tree sparrow, *Passer montanus* L. [40].

A peculiar phenomenon coming up in our results was the taxonomical (or only nomenclatural) confusion of *Ornithomya* species. Based on literature, traditional morphological identification of the smaller bird louse species in our study is *O. chloropus*, and the larger species *O. avicularia*. However, our own *O. chloropus* sequences matched most closely with the only *O. anchineura* in the public databases (EF531227), published by Petersen *et al*. [41]. Furthermore, the only two available *O. chloropus* sequences in the public databases (EF531213, KF453423) formed a separate cluster, matching 100 % to each other. Being beyond the scope of the current study, we leave this question open and call for future work to properly address this problem.

## Conclusions

Our study revealed relatively high prevalence for all the fly species in nests of *F. hypoleuca* and interesting spatial patterns in prevalence of the two louse flies. Our sample, however, did not indicate any major northward range shifts for the southern species as a consequence of warmer climate as compared to the information from the past. Taxonomy, differences in spatial prevalence of *Ornithomya* species and their roles in transmitting blood parasites call for further studies. For example, it would be interesting to compare blood parasite prevalence between the *Ornithomya* species. If the two *Ornithomya* species would differ in their rates of transmitting blood parasites, spatial gradient in their relative numbers could produce corresponding spatial gradients in blood parasite prevalence. Such spatial gradients in blood parasite prevalence have been previously reported (e.g. in Parids) relative to the distance to water bodies [42].

## Additional files

Additional file 1:
**Molecular analysis of puparia and adult bird louse flies.** Species and abundance of louse flies (*Ornithomya* spp.) and blowflies (*Protocalliphora* spp.) in nests of the pied flycatcher. (DOCX 46 kb) Additional file 2:
**Genetic distances between individual sequences.** (XLSX 18 kb)
