# Supplementary material for: Species and abundance of ectoparasitic flies (Diptera) in pied flycatcher nests in Fennoscandia
Source: Parasit Vectors. 2015 Dec 21;8:648. doi: 10.1186/s13071-015-1267-6 (PMC4687113; doi:10.1186/s13071-015-1267-6)
Supplement: Additional file 1: — Molecular analysis of puparia and adult bird louse flies. Species and abundance of louse flies (Ornithomya spp.) and blowflies (Protocalliphora spp.) in nests of the pied flycatcher. (DOCX 46 kb) [file 13071_2015_1267_MOESM1_ESM.docx]

**Species and abundance of louse flies (*Ornithomya* spp.) and blowflies (*Protocalliphora* spp.) in nests of the pied flycatcher**

Eeva T., Andersson T., Berglund Å., Brommer J., Hyvönen R., Klemola T., Laaksonen T., Loukola O., Morosinotto C., Rainio K., Sirkiä P., and Vesterinen E.J.

Supplement 1: The molecular analysis of pupae and adult bird louse flies

In order to confirm the morphological analysis of our samples, we conducted a DNA barcoding analysis using standard methods (Sorvari *et al.* 2012; Pilipenko *et al.* 2012). Only a subset of the samples collected for the study were used for molecular analysis. Besides our own samples, all the sequences representing the genera *Ornithomya* and *Protocalliphora* were downloaded from the GenBank, and the sequences representing the first half of the COI were included in the subsequent analysis. The trees including our own samples were drawn using software Geneious version 6.

The results show that our identification into species clusters is accurate, but there are some naming confusion between the sequences in the GenBank and the literature used for identification of these species (Figure S1.).





Figure S1. Molecular analysis for Ornithomya and Protocalliphora collected from *Ficedula hypoleuca* (Passeriformes, Muscicapidae) nets is Finland. A maximum-likelihood tree using General-time-reversible (GTR) substitution model is used for both groups. The consensus tree and branch support figures are drawn from 100 bootstrap replicates. For both trees, our own samples are denoted in bold-face text and the pupae are pointed by arrows. All the sequences for each species for the same gene region (first half of the COI) are shown with Genbank accession number. (a) ML tree for *Ornithomya*, and (b) ML tree *Protocalliphora*.

**References**

Geneious version 6.1 created by Biomatters. Available from ​http://www.geneious.com/​​

Kearse M, Moir R, Wilson A et al. (2012) Geneious Basic: an integrated and extendable desktop software platform for the organization and analysis of sequence data. Bioinformatics (Oxford, England), **28**, 1647–1649.

Pilipenko V, Salmela J, Vesterinen E (2012) Description and DNA barcoding of Tipula (*Pterelachisus*) recondita sp. n. from the Palaearctic region (Diptera, Tipulidae). ZooKeys, **192**, 51–65.

Sorvari J, Härkönen S, Vesterinen EJ (2012) First record of an indoor pest sawtoothed grain beetle *Oryzaephilus surinamensis* (Coleoptera: Silvanidae) from wild outdoor wood ant nest. Entomologica Fennica, **23**, 69–71.
